# The incidence of medically-attended norovirus gastro-enteritis in Japan: Modelling using a medical care insurance claims database

**DOI:** 10.1371/journal.pone.0195164

**Published:** 2018-03-30

**Authors:** Chia-Hsien Chang, Motonobu Sakaguchi, John Weil, Thomas Verstraeten

**Affiliations:** 1 Global Patient Safety Evaluation Japan, Takeda Development Center Japan, Takeda Pharmaceutical Company Limited, Osaka, Japan; 2 Takeda Pharmaceuticals International AG, Zurich, Switzerland; 3 P95 Pharmacovigilance and Epidemiology, Heverlee, Belgium; University of Liverpool, UNITED KINGDOM

## Abstract

**Background:**

The burden of medically-attended acute gastro-enteritis (MA-AGE) that can be attributed to norovirus is not well established in Japan. Using a nationwide database of medical care insurance claims, we estimated the incidence of medically-attended norovirus-attributable gastroenteritis (MA-NGE) in Japan.

**Methods:**

The incidences of MA-NGE outpatient consultations or hospitalization in Japan were modelled on seasonal patterns of MA-AGE for unspecified causes derived from the Japan Medical Data Center (JMDC) database for the period July 2007 to June 2015.

**Results:**

Mean age-adjusted annual incidence rates (per 10,000 person-years) of MA-NGE associated with outpatient care or hospitalization were 389 (95% CI 269–558) and 13 (95% CI 9–20), respectively. Highest rates were in children under 5 years of age: 1,569 (95% CI 1,325–1,792) for outpatient consultations and 48 (95% CI 39–56) for hospitalizations. Of all gastroenteritis episodes associated with outpatient care or hospitalization, 29% and 31% were attributed to norovirus, respectively. Norovirus was estimated to be responsible for 4,964,000 outpatient visits (95% CI 3,435,000–7,123,000) and 171,000 hospitalizations (95% CI 110,000–251,000) per year across Japan.

**Conclusions:**

Incidence rates of MA-AGE are high in Japan, and norovirus-attributable disease is at least as high as in some other developed countries.

## Introduction

Norovirus is the leading cause of acute gastroenteritis (AGE) worldwide, causing both community-acquired disease [1, 2] and healthcare-associated outbreaks [3, 4]. Incidence rates are higher in young children, and hospitalization is most common in the very young and the old [5]. Diagnostic testing for norovirus is generally only performed during gastroenteritis outbreaks and rarely performed routinely. Therefore, information on the burden of medically-attended norovirus-attributable gastroenteritis (MA-NGE) in the wider population is derived mainly through surveillance and prospective cohort studies.

In Japan the Infectious Disease Surveillance Center (IDSC) of the National Institute of Infectious Diseases (NIID) monitors the burden of norovirus-attributable AGE through 3,000 paediatric sentinel clinics under the National Epidemiological Surveillance of Infectious Diseases (NESID), and through data collected from outbreak-associated notifications [6],[7]. In addition, a number of studies have reported on the occurrence of norovirus-attributable disease based on convenience samples obtained from paediatric outpatient or hospitalized populations and in specific geographical settings of Japan [8–32], as reviewed in Thongprachum et al [32]. However, the findings of these studies are highly variable, ranging from 8% to 40% of AGE cases as being positive for norovirus, with smaller-sized studies generally reporting higher proportions. Hence, there is insufficient information on the prevalence or incidence of MA-NGE across the general population of all ages in Japan.

Studies in the US [33, 34] and recently in England [35] have used electronic healthcare databases and applied modelling techniques to estimate the burden of medically-attended gastroenteritis caused by norovirus at a population level. A medical administrative database in Japan that would be suitable for such an exercise is the Japan Medical Data Center (JMDC) database. The JMDC database is a nationwide databank of insurance claims capturing de-identified individual medical information from insured persons in Japan, including information on medically-attended acute gastroenteritis (MA-AGE). The aim of this study was to assess the incidence of MA-AGE and model the incidence of MA-NGE associated with outpatient consultancies or hospitalizations across all ages and for different age groups in the period 1 July 2007 to 30 June 2015 in Japan using the JMDC database.

## Materials and methods

### Data sources

Data were extracted from the JMDC database that contains de-identified individual medical information derived from insurance claims from various public and private healthcare plans in Japan [36]. The JMDC database expanded from a database of 330,000 persons in 2005 to include data from approximately 4.7 million individuals in Japan in 2015. To enable use of the database for research purposes, a secondary database is generated by linking files using of an anonymous identifier. As the JMDC database is an employee-based database and does not capture information from individuals and their dependents once they retire, the population in Japan aged 65 years and older is underrepresented in the database.

### Study design and cohort

Since data from before 2007 are relatively limited, we used a retrospective cohort design, extracting data collected during the eight year period between July 2007 and June 2015 to estimate the incidence of MA-AGE and model the incidence of MA-NGE. Data were included from patients who had at least 180 days of previous continuous enrolment recorded in the database between January 2007 and June 2015.

### Ethical considerations

Since the data used for estimation is the number of occurrences of MA-AGE, the privacy and confidentiality of the data are well-protected. Although patients can be identified via a unique identifier by JMDC, the secondary database used for research purposes has de-identified data only.

### Case definition

ICD-10 codes were used to extract MA-AGE events from the database (S1 Table). Events were included when any ICD-10 code relating to gastroenteritis, gastrointestinal infection or a gastrointestinal pathogen was recorded, regardless of whether this was a primary, secondary or any other positioned diagnosis. Outpatient care visits included visits to primary care providers and ambulatory clinics in hospitals, including emergency room visits. Data on hospitalization associated with a gastroenteritis-related diagnosis were extracted from discharge records. Outpatient visits and hospitalizations were counted separately: if a patient was first seen in an outpatient care setting and later hospitalized, this was counted as two separate episodes in the respective outpatient and hospitalization analyses. When patients had more than one recorded outpatient or hospitalization associated gastroenteritis event, this was considered a new episode if the time between the two recorded events was at least 30 days.

Episodes were grouped into cause-unspecified or cause-specific episodes, with the latter being further categorized into norovirus, rotavirus, *Clostridium difficile*, other bacterial cause, or parasitic cause.

### Statistical methods

Observed incidence rates per 10,000 person-years and exact Poisson 95% confidence intervals were calculated for the total population, as well as by age group, and time (year, month). Age-adjusted incidence rates were calculated by adjusting the age distribution in the database proportionally to age group specific census data for the population in Japan.

Since only a small proportion of episodes are cause-specified, the proportion attributable to norovirus was estimated using an indirect modelling method previously developed and published for US healthcare databases [33, 34], with minor adaptations. Briefly, the model assumes that among the episodes with unspecified cause, the number due to a specified pathogen or pathogen group in a given month is proportional to the number of episodes actually coded for that same pathogen in the month. A Poisson regression model with an identity link for each month of a season was applied to predict the number of cause-unspecified MA-AGE episodes. Separate models were fitted for each age group *x* and health care setting y. The expected number (E) of cause-unspecified episodes in a month was modelled as a function of recorded number (N) of episodes attributed to rotavirus, *C*. *difficile*, other bacterial causes and parasitic causes in the same month using the formula:

$$E_{cause-unspecified}=\alpha +(\beta_{1}\times N_{rotavirus\;0-4years,y})+(\beta_{2}\times N_{C.difficile\;x,y})+(\beta_{3}\times N_{other\;bacterial\;x,y})+(\beta_{4}\times N_{parasitic\;x,y})+\gamma \times {Time}_{y}$$ (1)

The β coefficient represents the relative contribution of each specified pathogen, and the intercept α represents the number of background visits that are not explained by an infection due to one of the specified pathogens categories. Secular trends were controlled for by including a sequential variable for month of study, Time, and secular time trends of unspecific visits, γ. In line with previous studies, the number of rotavirus-attributable episodes for all age groups was calculated using data from children aged 0 to 4 years due to lack of data in other age groups, multiplying the rate in the age group 0 to 4 years with the denominator in the respective age groups. The number of norovirus-attributable gastroenteritis visits for each month was the model residual (or variance remained unexplained) of that month minus the minimum monthly residual of the season. Finally, the coded norovirus-attributable number of episodes in each month was added to the estimated counts of norovirus episodes in that month to get the overall monthly estimates. The model assumed a negative binomial distribution to account for over-dispersion.

Descriptive statistics, including means (standard deviation, SD) for continuous variables and number (percentage, %) for category variables were used to describe baseline characteristics. Confidence intervals for the estimated norovirus counts were obtained by bootstrapping based on Poisson distribution of means from the original data using 1,000 replicates. All statistical analyses were carried out using SAS software 9.3 (SAS Institute Inc., 2014, Cary North Carolina, USA).

## Results

The study population comprised 4,218,452 individuals who had at least 180 days enrolment registered in the JMDC database, of which 53.7% were male. At the time of enrolment, 9.5% of the population was under 5 years of age, 15.2% was 5–17 years old, 73.9% was 18–64 years old, and 2.3% was aged 65 years and older.

### Incidence of MA-AGE according to healthcare, age, and specified cause

In the eight-year period from July 2007 to June 2015, a total of 2,041,904 MA-AGE episodes associated with outpatient care and 43,424 episodes associated with hospitalization were recorded in the JMDC database, corresponding to mean age-adjusted annual incidence rates of 1,355 (95% CI 1,158–1,629) per 10,000 person-years for MA-AGE episodes associated with outpatient care, and 42 (95% CI 36–50) per 10,000 person-years for MA-AGE episodes associated with hospitalization. Mean annual incidence rates for different age groups are shown in Table 1. Children less than 5 years of age had the highest incidence rates of both outpatient care and hospitalization associated MA-AGE, while the age group of 65 years and older had the second highest rates for hospitalization-associated episodes.

**Table 1:** Mean annual incidence rates of MA-AGE associated with outpatient care or hospitalization according to age groups.

	Outpatient Care	Hospitalization
	Incidence rate (per 10,000 person-years) (95% CI)	
**0–4 years**	**7,090** (6,635–7,634)	**187** (154–224)
**5–17 years**	**2,587** (2,312–2,974)	**34** (31–38)
**18–64 years**	**1,043** (896–1,249)	**23** (21–26)
**≥ 65 years**	**532** (295–878)	**69** (55–86)
All*	**1,355** (1,158–1,629)	**42** (36–50)

* Data is adjusted for mean age distributions in the Japan census population for the period 2007–2015.

Pathogen-specified and unspecified episode counts were derived from coded gastroenteritis visits in the JMDC database for the period July 2007 to June 2015. An infectious cause was specified for 29% of the hospitalization-associated MA-AGEs episodes and only 3% of the outpatient care-associated episodes in the database. Bacterial infections other than *C*. *difficile* was the most frequently specified group of pathogens associated with MA-AGE and rotavirus the most frequently specified single pathogen (Table 2).

**Table 2:** Mean annual number and incidence rates of cause-specified and unspecified MA-AGE episodes associated with outpatient care or hospitalization.

	Outpatient care		Hospitalization	
	Episodes (n)	Incidence rates Per 10,000 person-years (95% CI)	Episodes (n)	Incidence rates Per 10,000 person-years (95% CI)
**Cause-unspecified**	247,372	1531.1 (1464.7–1888.4)	3,869	23.95 (23.13–29.13)
**Rotavirus**	1,650	10.21 (9.38–13.08)	541	3.35 (2.97–5.21)
***C*. *difficile***	36	0.22 (0.20–0.26)	185	1.14 (1.05–1.21)
**Other bacterial**	5,340	33.05 (31.52–45.39)	647	4.00 (3.86–5.13)
**Parasitic**	29	0.18 (0.16–0.25)	14	0.08 (0.07–0.10)
**Norovirus**	812	5.02 (3.08–5.65)	173	1.07 (0.51–1.22)

Pathogen-specified and unspecified episode counts were derived from coded gastroenteritis visits in the JMDC database for the period July 2007 –June 2015

**Fig 1:**
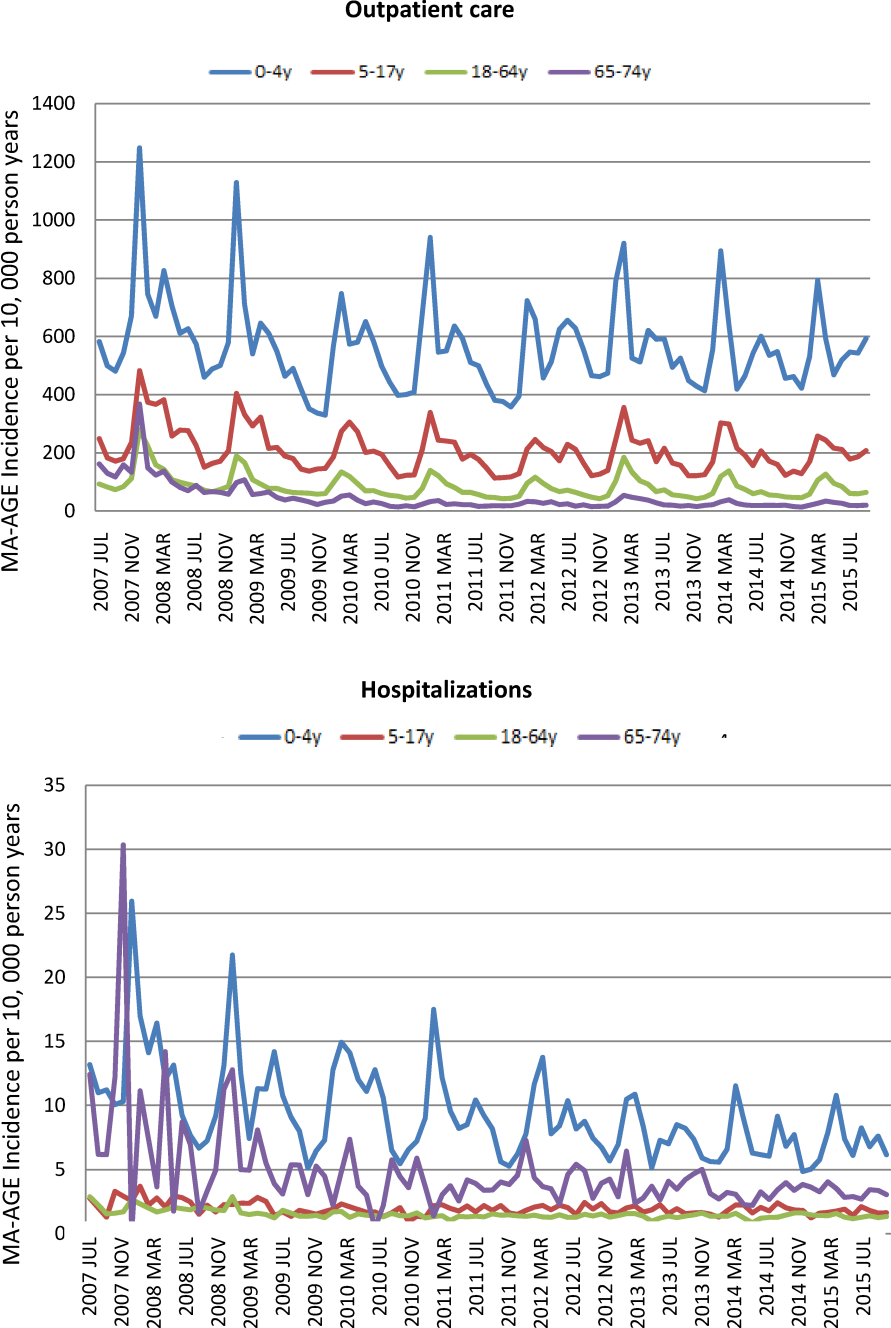
Seasonality of cause-unspecified MA-AGE by age group associated with outpatient care and or hospitalizations. 3-monthly mean incidence rates of cause-unspecified MA-AGE episodes in the JMDC database for the period July 2007 –June 2015 were calculated for different age groups.

Clear seasonal patterns with regular peaks in the early winter were observed for cause-unspecified outpatient care AGE across all age groups, and for hospitalization-associated episodes in young children and the elderly (Fig 1). This observation of seasonality supports the applicability of using the indirect model to estimate incidence rates of norovirus disease.

### Modelled incidence rates of medically attended norovirus gastroenteritis (MA-NGE)

**Fig 2:**
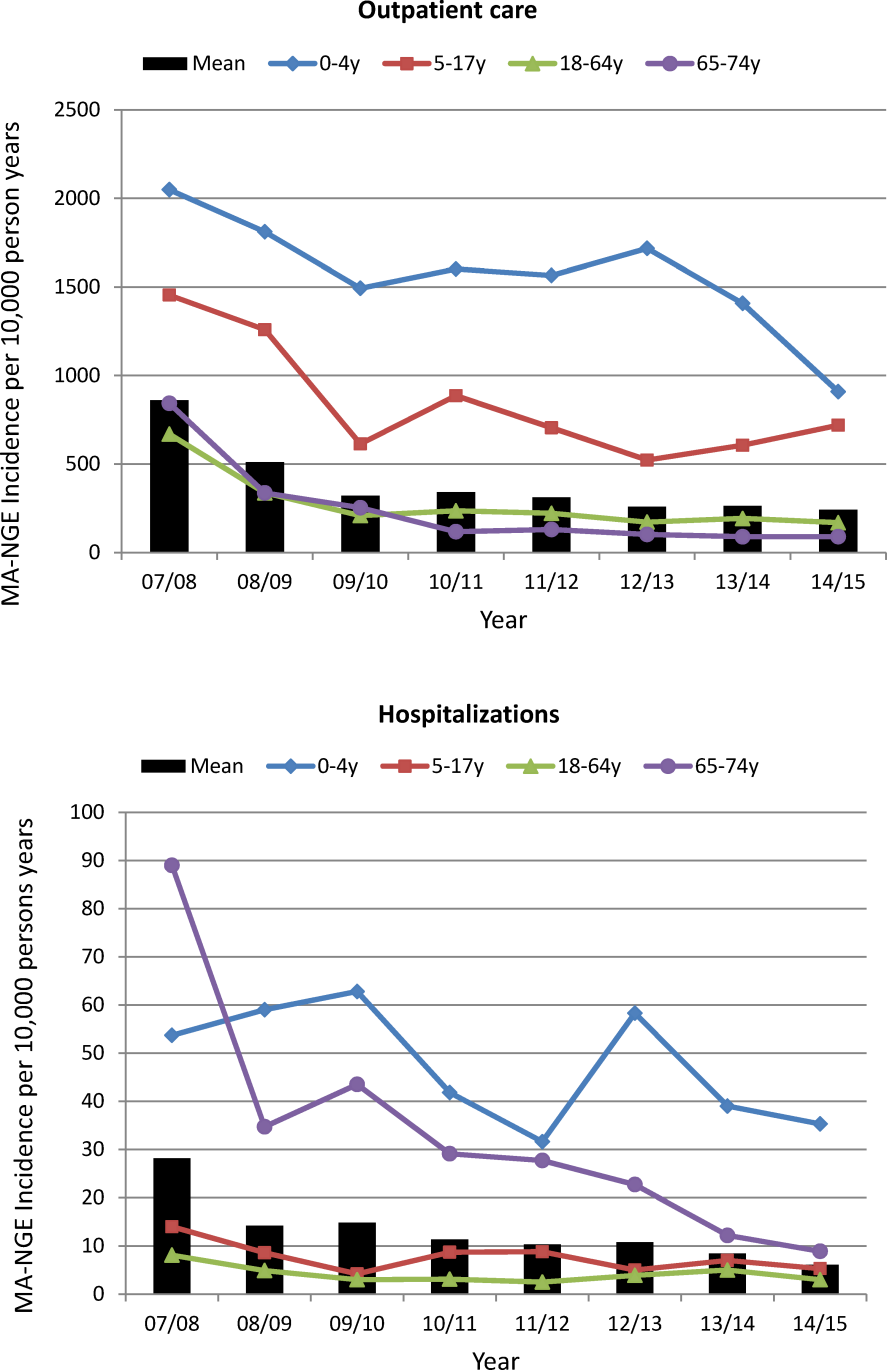
Annual incidence rates of MA-NGE associated with outpatient care or hospitalizations. Annual rates of MA-NGE associated with outpatient care or hospitalizations were estimated from the JMDC database using the indirect regression model and adjusted for year-specific age distributions in the Japan census population for the period 2007–2014.

Annual age-adjusted incidence rates of MA-NGE associated with outpatient care or hospitalization for the eight-year study period in Japan were estimated using the residual regression model (Fig 2). Outpatient care-associated incidence rates of MA-NGE were the highest in the first year of the study (July 2007—June 2008) for all age groups, and the same was found for hospitalization-associated episodes, in particular in the age group of 65 years and older.

The age-adjusted mean annual incidence rate of MA-NGE associated with outpatient care was estimated at 389 (95% CI 269–558) cases per 10,000 person-years and for MA-NGE associated with hospitalization at 13 (95% CI 9–20) cases per 10,000 person-years. This corresponds with 29% of all MA-AGE outpatient care visits and 31% of all MA-AGE related hospitalizations being attributed to norovirus.

Table 3 shows estimated mean annual incidence rates of MA-NGE associated with outpatient care or hospitalization for different age groups. Children under 5 years of age had the highest rates of MA-NGE associated with outpatient care (mean annual incidence rate of 1,569/10,000 person-years; 95% CI 1,325–1,792) and hospitalization (mean annual incidence rate of 48/10,000 person-years; 95% CI 39–56). The incidence of hospitalization-associated MA-NGE was the second highest in the age group of 65 years and older (mean annual incidence rate of 34/10,000 person-years; 95% CI 19–53).

**Table 3:** Estimated number and mean annual incidence rates of MA-NGE in relation to health care setting and age group in Japan.

	Outpatient care		Hospitalizations	
	Incidence rates Per 10,000 person-years (95% CI)	Episodes ^a^ N × 1,000 (95% CI)	Incidence rates Per 10,000 person-years (95% CI)	Episodes ^a^ N × 1,000 (95% CI)
0–4 years	1,569 (1,325–1,792)	834 (705–953)	48 (39–56)	25 (21–30)
5–17 years	845 (639–1,101)	1,274 (963–1,660)	8 (6–10)	12 (8–15)
18–64 years	275 (189–412)	2,127 (1,456–3,182)	4 (3–6)	32 (24–43)
≥ 65 years	245 (107–445)	734 (319–1,332)	34 (19–53)	100 (56–160)
All	389 (269–558)	4,964 (3,435–7,123)	13 (9–20)	171 (110–251)

The number and mean annual incidence rates of MA-NGE episodes in relation to health care setting and age group for Japan for the period July 2007 –June 2015 were estimated using the residual model applied to the JMDC database: E_cause-unspecified = α + (β_1_ × N_rotavirus_0-4years,y_) + (β_2_ ×N_*C*.*difficile*_x,y_) + (β_3_ × N_other bacterial_x,y_) + (β_4_ × N_parasitic_x,y_) + γ × Time_y_.

a) Numbers (expressed as thousand population; x 1,000) were estimated based on the annual rate calculated in the JMDC population extrapolated to age group specific census data for the Japanese population

**Fig 3:**
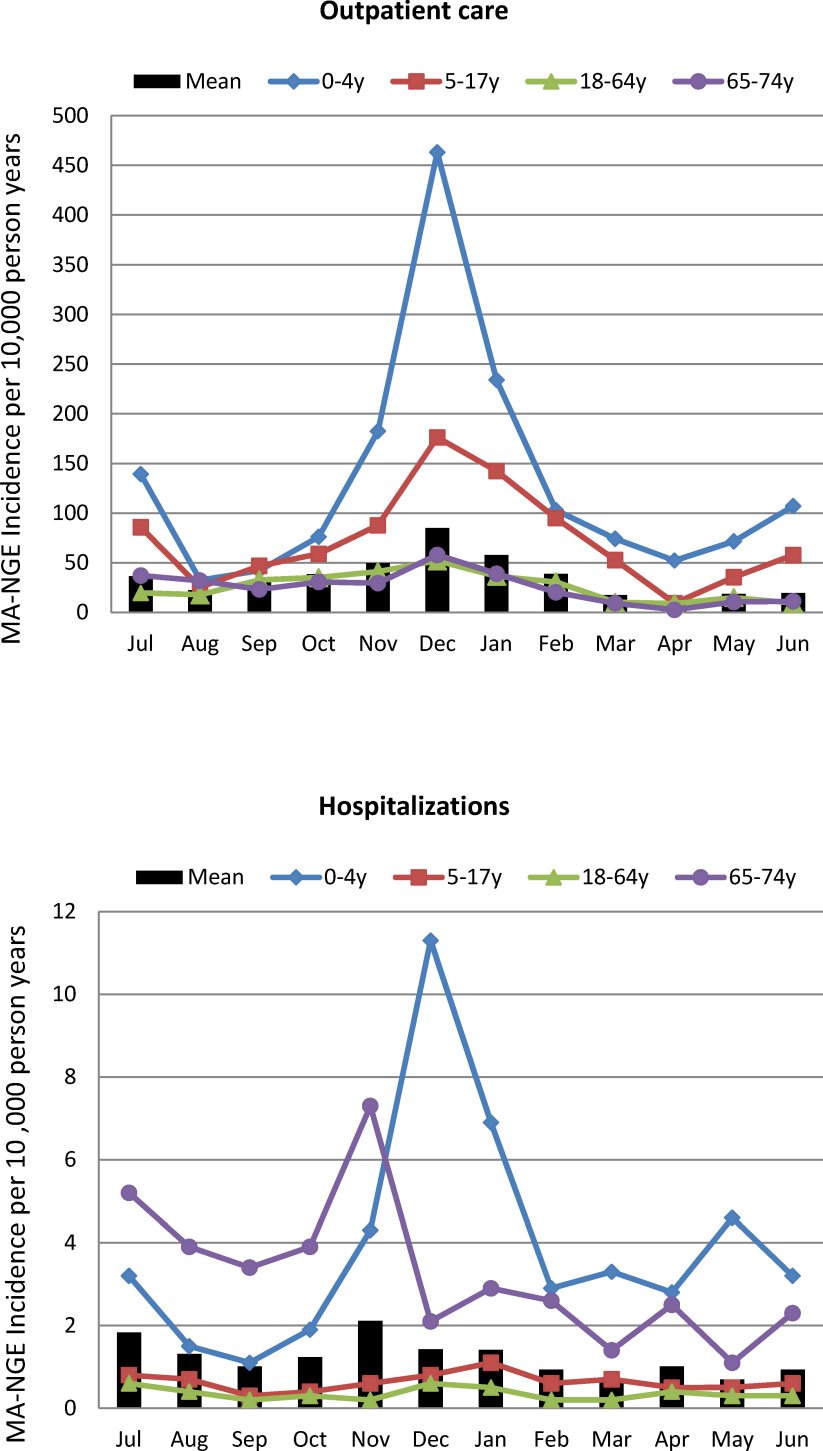
Monthly mean rates of MA-NGE associated with outpatient visits or hospitalizations for all ages and different age groups. Overall and age-group specific mean monthly incidence rates of MA-NGE were estimated from the JMDC database using the indirect regression model and adjusted for year-specific age distributions in the Japan census population in the period 2007–2014.

Plotting estimated incidence rates of MA-NGE for different months of the year, episodes were found to peak in the winter months November, December and January (Fig 3). In December, mean incidence rates of MA-NGE in children younger than 5 years of age were estimated to be as high as 463/10,000 (95% CI 235–602) for episodes associated with outpatient care, and 11/10,000 (95% CI 8–16) for episodes associated with hospitalization. On average, 59% of all outpatient visits and 44% of all hospitalizations attributed to norovirus throughout the year occurred between November and February.

### Estimated annual number of MA-NGE related outpatient visits and hospitalizations in Japan

By extrapolating incidence rates modelled from the JMDC database to the Japanese national population using age-group specific census data, norovirus was estimated to be responsible for 4,964,000 (95% CI 3,435,000–7,123,000) outpatient visits and 171,000 (95% CI 110,000–251,000) hospitalizations per year across Japan (Table 3).

### Estimated proportion of MA-AGE due to rotavirus

In addition to the above estimates for norovirus, we also estimated that rotavirus caused 16% of outpatient visits for AGE and 25% of hospitalizations for AGE, corresponding to a total of 317,599 outpatient visits and 10,059 hospitalizations. The proportions per age group were 16%, 23%, 13%, and 20% for outpatients and 30%, 27%, 21% and 25% for hospitalization in the 0–4, 5–17, 18–64 and 65+ age groups, respectively.

## Discussion

In Japan trends in the epidemiology of AGE are assessed by passive outbreak surveillance, complemented by active surveillance through 3,000 paediatric sentinel clinics distributed throughout Japan, with cases being tested for rotavirus, norovirus and sapovirus [6, 7]. The overall and norovirus-attributable burdens of MA-AGE across all age groups are therefore not well established in Japan. Using the JMDC database, we were able to estimate age group-specific mean annual incidence rates of MA-NGE in Japan for the period July 2007 to June 2015, finding mean annual incidence rates of 389 outpatient visits per 10,000 person-years and 13 hospitalization-related cases per 10,000 person-years across the total population. As reported for other countries, incidence rates were highest in children under 5 years of age [33–35].

Compared with other high-income countries, the incidence rates of MA-AGE and MA-NGE outpatient care visits were high in Japan. For example, studies based on an US insurance claims database and an UK administrative database report mean annual incidence rates of outpatients visits for MA-AGE of 459/10,000 person-years [34] and 290/10,000 person-years [35], respectively, compared with a rate of 1,355/10,000 person-years found in the JMDC database for Japan. Using the same modelling approach as applied here for Japan, these studies estimated incidence rates of MA-NGE associated with outpatient care at 57/10,000 person-years in the US [34] and 49/10,000 person-years in the UK [35], compared with an estimated rate of 389/10,000 person-years in this study for Japan. However, incidence rates of hospitalization-associated MA-AGE in Japan found in our analysis of the JMDC database (42/10,000 person-years) approached rates reported by these studies for the US (25/10,000 person-years) and England (56/10,000 person-years). A possible explanation for higher rates of MA-AGE outpatient consultations in Japan is a higher primary healthcare seeking behaviour of the Japanese population compared with the US and many other countries [37, 38], combined with a auniversal health care system that provides affordable health care services to all persons. We are not aware of any similar assessment having been performed for Japan. Paediatric infectious gastroenteritis, including norovirus infections, is monitored in Japan through 3000 sentinel paediatric practices [39], which report approximately one million cases of infectious gastroenteritis annually but do not give incidence rates [40]. Incidence rates of MA-NGE were the highest in the first year of the study (July 2007—June 2008), both for episodes associated with outpatient care and hospitalization, which is possibly explained by an ongoing elevated transmission of norovirus following the 2006–2007 norovirus outbreak in Japan [41].

The proportion of MA-AGE episodes that is attributable to norovirus is at least as high in Japan as in other developed countries. A systematic review by Ahmed et al. reported a prevalence of 20% (95% 17–22) for all developed countries included in the meta-analysis of studies published between 2008 and 2014 [42]. The review included 13 studies from Japan for which the average prevalence of norovirus-attributable disease can be calculated to be 21–28%, depending on the calculation method. In our study we found that 29% of MA-AGE episodes were attributable to norovirus: this slightly higher number could be explained to a certain extent by the JMDC database including information on all age groups (although older adults are under-represented), while the studies from Japan included in the systematic review were predominantly based on small paediatric outpatient populations, or hospitalised populations in specific geographical settings of Japan. The proportion of disease caused by norovirus therefore seems to be at the higher end of the spectrum for developed countries, but not exceptionally so as the variation amongst developed countries included in the review was high, from a low prevalence of 9% in the Netherlands to a high prevalence of 26% in Finland, comparable to that in Japan. Differences in food culture such as the consumption of raw seafood in Japan could contribute to a higher prevalence of norovirus and therefore provide the opportunity for food handlers to contaminate these foods. When a specific food is identified as the cause of a norovirus foodborne outbreak it is nearly always raw seafood. However, in over 80% of foodborne outbreaks no causative agent is identified, so the risk from raw seafood is not fully characterized [43].

Our study confirms that norovirus is an important cause of MA-AGE in Japan, not only in children as reported by the National Epidemiological Surveillance of Infectious Diseases (NESID) sentinel surveillance, but also in other age groups. In particular in the age group of 65 years and older the burden of hospital-related MA-NGE is high, with 34 out of 10,000 elderly being hospitalized with an indication for norovirus disease, compared with 50/10,000 children under the age of 5. This is in line with findings from the US and UK studies [35].

A few observations lend support to the validity of the residuals model used in our study. First, the finding that norovirus disease was seasonal in Japan and peaked in the winter months corresponds with other reports on norovirus infections based on surveillance data from Japan [44]. Secondly, our results show heightened activity of norovirus-attributable outpatient visits and hospitalizations in children under 5 years of age during the year 2012/2013, which accords with an epidemic of norovirus due to the emergence of a new norovirus genotype GII.4 variant that occurred in Japan in that period[45].

Our study also has a number of limitations. First, the number of elderly individuals included in the analysis is relatively small as the JMDC database is based on insurance claims sourced from the Japanese union-managed health insurance system (Health Insurance Association) and mainly includes data from people who are employed (and hence younger than 65 years old) by middle to large size companies and their dependents. The relatively small group of active working elderly for whom data are present in the JMDC database may therefore not be fully representative for people 65 years and older in Japan, and our study may therefore have overestimated or underestimated the true burden of norovirus disease in those aged 65–74 years old. Moreover, since people in Japan transfer to a different insurance program when they are 75 years and older, there was no information on this older age group that could be analysed for this study. Other studies have shown the highest rate of severe norovirus disease occurs in the very old of 75 years and older [46, 47]. Secondly, the residuals modelling method that we used to estimate the burden of norovirus assumes that all cause unspecified MA-AGE episodes in the database are due to norovirus. This may result in overestimating the true rates of norovirus-attributable gastroenteritis [33].

In summary, our study provides new data on the burden of MA-AGE and MA-NGE across all age groups in Japan, showing that the incidence of MA-AGE is high in Japan, being consistent to that reported in surveillance data, and that the proportion of disease attributable to norovirus is at least as high as in some other developed countries. Secondly, our study supports findings from other studies that administrative datasets containing population-based information on primary care and hospitalization in a country can be used to estimate and study trends of the burden of MA-AGE and MA-NGE in the absence of routine diagnostic testing.

## Supporting information

S1 Table Pathogen categories and diagnostic codes used to identify acute gastroenteritis in the JMDC database. (DOCX)

S2 Table Age group specific census data for the Japanese population during study period. (DOCX)
