# Supplementary material for: The incidence of medically-attended norovirus gastro-enteritis in Japan: Modelling using a medical care insurance claims database
Source: PLoS One. 2018 Mar 30;13(3):e0195164. doi: 10.1371/journal.pone.0195164 (PMC5877878; doi:10.1371/journal.pone.0195164)
Supplement: S1 Table — (DOCX) [file pone.0195164.s001.docx]

# **Supplementary Table 1. Pathogen categories and diagnostic codes used to identify acute gastroenteritis in the JMDC database**

| **Pathogen** | **ICD10 code** | **Japanese domestic disease code** | **Description** |
| --- | --- | --- | --- |
| Norovirus | A081 | 0788006 | Epidemic vomiting |
| Norovirus | A081 | 8842417 | Norovirus gastroenteritis |
| Norovirus | A081 | 8842528 | Norovirus enteritis |
| Rotavirus | A080 | 0088004 | Rotaviral infection |
| Rotavirus | A080 | 0088005 | Rotaviral enteritis |
| Rotavirus | A080 | 8847684 | Rotaviral gastroenteritis |
| *C. difficile* | A047 | 5580020 | Pseudomembranous colitis |
| *C. difficile* | A047 | 8844165 | Pseudomembranous colitis |
| *C. difficile* | A047 | 8847089 | Enterocolitis due to Clostridium difficile |
| Bacterial | A000 | 0010001 | Classical cholera |
| Bacterial | A000 | 8830244 | Asia cholera |
| Bacterial | A009 | 0019001 | Cholera |
| Bacterial | A010 | 0020002 | Infection due to Salmonella typhi |
| Bacterial | A011 | 0021001 | Paratyphoid fever A |
| Bacterial | A014 | 0029001 | Paratyphoid fever |
| Bacterial | A020 | 0030001 | Salmonella enteritis |
| Bacterial | A020 | 0030002 | Salmonella enteritis |
| Bacterial | A020 | 0030003 | Salmonella enteritis |
| Bacterial | A029 | 0030004 | Foodborne intoxication by Salmonella |
| Bacterial | A029 | 8834040 | Salmonella infection |
| Bacterial | A033 | 8836851 | Shigellosis due to Shigella sonnei |
| Bacterial | A039 | 0049005 | Shigellosis |
| Bacterial | A040 | 8839554 | Enteropathogenic Escherichia coli infection |
| Bacterial | A041 | 8837686 | Enterotoxigenic Escherichia coli infection |
| Bacterial | A043 | 8837683 | Enterohaemorrhagic Escherichia coli infection |
| Bacterial | A044 | 0058003 | Foodborne intoxication by Escherichia coli |
| Bacterial | A044 | 8837369 | Enteritis due to Escherichia coli. |
| Bacterial | A045 | 8831672 | Campylobacter enteritis |
| Bacterial | A046 | 8842304 | Enteritis due to Yersinia enterocolitica |
| Bacterial | A048 | 8830120 | Enteritis due to Methicillin-resistant Staphylococcus aureus |
| Bacterial | A048 | 8832848 | Enteritis due to Klebsiella pneumoniae |
| Bacterial | A048 | 8832854 | Enteritis due to Clostridium welchii |
| Bacterial | A048 | 8847268 | Enteritis due to Pseudomonas aeruginosa |
| Bacterial | A049 | 0085003 | Bacterial colitis |
| Bacterial | A049 | 0085004 | Bacterial enteritis |
| Bacterial | A049 | 0085005 | Bacterial gastroenteritis |
| Bacterial | A049 | 8833937 | Bacterial diarrhea |
| Bacterial | A050 | 0050002 | Foodborne staphylococcal intoxication |
| Bacterial | A051 | 8840201 | foodborne intoxication due to Clostridium botulinum |
| Bacterial | A051 | 8848219 | Infant botuilsm |
| Bacterial | A052 | 0052004 | Foodborne Clostridium perfringens intoxication |
| Bacterial | A052 | 8830865 | Enteritis necroticans |
| Bacterial | A053 | 0054001 | Foodborne Vibrio parahaemolyticus intoxication |
| Bacterial | A053 | 0054003 | Vibrio parahaemolyticus infection |
| Bacterial | A054 | 8836060 | Foodborne Bacillus cereus intoxication |
| Bacterial | A058 | 0058007 | Foodborne intoxication by Yersinia enterocolitica |
| Bacterial | A059 | 0059002 | Bacterial foodborne intoxication |
| Bacterial | A183 | 8833046 | Diarrhea, tuberculosis-related |
| Parasitical | A060 | 0060001 | Acute amoebic dysentery |
| Parasitical | A060 | 0069002 | Amoebic dysentery |
| Parasitical | A060 | 8832274 | Acute amoebiasis |
| Parasitical | A060 | 8837669 | Intestinal amoebiasis |
| Parasitical | A061 | 0061002 | Chronic amoebic dysentery |
| Parasitical | A061 | 8840374 | Chronic intestinal amoebiasis |
| Parasitical | A062 | 0062001 | Amoebic nondysenteric colitis |
| Parasitical | A069 | 8830309 | Amoebiasis |
| Parasitical | A071 | 8835148 | Giardiasis |
| Parasitical | A071 | 8840928 | Lambliasis |
| Parasitical | A072 | 8844039 | Cryptosporidium diarrhea |
| Parasitical | A078 | 8837759 | Intestinal trichomoniasis |
| Parasitical | A079 | 8833297 | Protozoal colitis |
| Unspecified | A082 | 8830271 | Adenoviral enteritis |
| Unspecified | A083 | 8830928 | Enterovirus enteritis |
| Unspecified | A084 | 0088002 | Viral gastroenteritis |
| Unspecified | A084 | 0088003 | Viral enteritis |
| Unspecified | A084 | 8830750 | Viral diarrhea |
| Unspecified | A085 | 0088007 | Contagious diarrhea |
| Unspecified | A09- | 0090001 | Infectious gastroenteritis |
| Unspecified | A09- | 0090002 | Infectious colitis |
| Unspecified | A09- | 0090003 | Infectious enteritis |
| Unspecified | A09- | 0090005 | Stomach flu (stomach and intestines) |
| Unspecified | A09- | 0090006 | Stomach flu (large intestines) |
| Unspecified | A09- | 0090007 | Stomach flu (intestines) |
| Unspecified | A09- | 0091002 | Catarrhal gastroenteritis |
| Unspecified | A09- | 0091005 | Gastroenteritis |
| Unspecified | A09- | 0091007 | Ileitis |
| Unspecified | A09- | 0091009 | Acute gastroenteritis |
| Unspecified | A09- | 0091012 | Acute colitis |
| Unspecified | A09- | 0091014 | Acute enteritis |
| Unspecified | A09- | 0091021 | Colitis |
| Unspecified | A09- | 0091022 | Catarrhal enteritis |
| Unspecified | A09- | 0091023 | Enteritis |
| Unspecified | A09- | 0093012 | Refractory Infant diarrhea |
| Unspecified | A09- | 0093016 | Diarrhea with milky white stool |
| Unspecified | A09- | 0093021 | Infant diarrhea |
| Unspecified | A09- | 5580001 | Sigmoiditis |
| Unspecified | A09- | 5789014 | Hemorrhagic enteritis |
| Unspecified | A09- | 5789018 | Hemorrhagic colitis |
| Unspecified | A09- | 8831621 | Infectious diarrhea |
| Unspecified | A09- | 8833267 | Diarrhea |
| Unspecified | A09- | 8838431 | Infant diarrhea in winter |
| Unspecified | J118 | 7890004 | Stomach flu |
| Unspecified | J118 | 8830721 | Influenza gastroenteritis |
| Unspecified | K529 | 5580006 | Chronic colitis |
| Unspecified | K529 | 5580021 | Habitual diarrhea |
| Unspecified | K529 | 5580033 | Chronic gastroenteritis |
| Unspecified | K529 | 5580037 | Chronic enteritis |
| Unspecified | K529 | 8838959 | fermentation diarrhea |
| Unspecified | K529 | 8839188 | Noninfective Sigmoiditis |
| Unspecified | K529 | 8839189 | Noninfective gastroenteritis |
| Unspecified | K529 | 8839190 | Noninfective Ileitis |
| Unspecified | K529 | 8839192 | Noninfective Jejunitis |
| Unspecified | K529 | 8839193 | Noninfective diarrhea |
| Unspecified | K529 | 8839194 | Noninfective colitis |
| Unspecified | K529 | 8839195 | Noninfective enteritis |
| Unspecified | K529 | 8840341 | Chronic diarrhea |
| Unspecified | K529 | 8845790 | Enteritis |
| Unspecified | P783 | 0093007 | Neonatal diarrhea |
