# Supplementary material for: The incidence of medically-attended norovirus gastro-enteritis in Japan: Modelling using a medical care insurance claims database
Source: PLoS One. 2018 Mar 30;13(3):e0195164. doi: 10.1371/journal.pone.0195164 (PMC5877878; doi:10.1371/journal.pone.0195164)
Supplement: S2 Table — (DOCX) [file pone.0195164.s002.docx]

Supplementary Table 2. Age group specific census data for the Japanese population during study period

| **Year** | **2007** | **2008** | **2009** | **2010** | **2011** | **2012** | **2013** | **2014** | **2015** | **Mean** |
| --- | --- | --- | --- | --- | --- | --- | --- | --- | --- | --- |
| 0-4y | 5,434 | 5,405 | 5,376 | 5,309 | 5,302 | 5,273 | 5,239 | 5,214 | 5,041 | 5,319 |
| 5-17y | 15,522 | 15,386 | 15,243 | 15,187 | 15,037 | 14,873 | 14,727 | 14,591 | 14,157 | 15,071 |
| 18-64y | 79,352 | 78,686 | 77,884 | 78,077 | 77,709 | 76,574 | 75,433 | 74,274 | 70,827 | 77,249 |
| 65-74y | 14,761 | 14,997 | 15,295 | 15,291 | 15,043 | 15,601 | 16,298 | 17,083 | 17,150 | 15,546 |
| 75y- | 12,704 | 13,220 | 13,711 | 14,193 | 14,707 | 15,191 | 15,601 | 15,916 | 16,090 | 14,405 |

Unit: 1,000 person

Resource：adapted by Portal site of Official Statistic of Japan (<http://www.e-stat.go.jp/>)
